# Circadian rhythms in insecticide susceptibility, metabolic enzyme activity, and gene expression in *Cimex lectularius *(Hemiptera: Cimicidae)

**DOI:** 10.1371/journal.pone.0218343

**Published:** 2019-06-17

**Authors:** Muhammad Fazli Khalid, Chow-Yang Lee, Stephen L. Doggett, G. Veera Singham

**Affiliations:** 1 Centre for Chemical Biology, Universiti Sains Malaysia, Bayan Lepas, Penang, Malaysia; 2 Urban Entomology Laboratory, Vector Control Research Unit, School of Biological Sciences, Universiti Sains Malaysia, Minden, Penang, Malaysia; 3 Department of Medical Entomology, NSW Health Pathology, Westmead Hospital, Westmead, NSW, Australia; Institute of Zoology Chinese Academy of Sciences, CHINA

## Abstract

Many insect species display daily variation of sensitivity to insecticides when they are exposed to the same concentration at different times during the day. To date, this has not been investigated in bed bugs. To address this, we explored circadian rhythms in insecticide susceptibility, xenobiotic metabolizing (XM) gene expressions, and metabolic detoxification in the common bed bug, *Cimex lectularius*. An insecticide susceptible Monheim strain of *C*. *lectularius* was most tolerant of deltamethrin during the late photophase at ZT9 (i.e. nine hours after light is present in the light-dark cycle (LD) cycle) and similarly repeated at CT9 (i.e. nine hours into the subjective day in constant darkness (DD)) suggesting endogenous circadian involvement in susceptibility to deltamethrin. No diel rhythm was observed against imidacloprid insecticide despite significant daily susceptibility in both LD and DD conditions. Rhythmic expressions of metabolic detoxification genes, *GSTs1* and *CYP397A1* displayed similar expression patterns with total GST and P450 enzyme activities in LD and DD conditions, respectively. The oscillation of mRNA levels of *GSTs1* and *CYP397A1* was found consistent with peak phases of deltamethrin susceptibility in *C*. *lectularius*. This study demonstrates that circadian patterns of metabolic detoxification gene expression occur within *C*. *lectularius*. As a consequence, insecticide efficacy can vary dramatically throughout a 24 hour period.

## Introduction

Bed bug infestations are on the rise and these insects have now re-emerged as important public health pests globally [1]. The common bed bug, *Cimex lectularius* L., more prevalent in temperate regions, and the tropical bed bug, *Cimex hemipterus* (F.), more prevalent in tropical and sub-tropical regions, are the two bed bug species that feed preferentially on human [2,3]. Bed bug infestations reduce the quality of lives among the general public. The bite may result in various clinical manifestations, including mild to severe allergic reactions, such as itchiness, erythematous rash or urticaria, and occasional systemic reactions [4–7]. Bed bugs were nearly eradicated during the mid-1900s in developed nations, largely due to the liberal and widespread use of insecticides such as DDT [5,8]. Nonetheless, reservoir populations persisted in developing nations [1] and over the past two decades, there has been a global resurgence of bed bugs across the world [1,5]. The resurgence of bed bugs has been associated with various factors, including the increase in global travel, changes in pest control practices, but most importantly, the evolution of insecticide resistance [1,5,9–12].

Bed bug eradication is complicated, time consuming, and expensive. Integrated pest management is normally recommended in bed bug management and may encompass the use of non-chemical options such as lethal temperatures (extreme cold or heat), vacuuming, and the direct removal of infested items [13], as well as the judicious use of insecticides [8]. Of these, insecticide treatment remains the most convenient and cost effective method used to eradicate bed bugs infestations [5,8,11], although is often unreliable due to issues of insecticide resistance [12].

Insecticide toxicity on insects may be influenced by numerous physical, chemical, or pathophysiological factors [14]. Beck [15] was the first to demonstrate that insect mortality caused by an insecticide may be influenced by the time of day at which it was applied. This was observed in the German cockroach, *Blatella germanica* L. that displayed rhythmic susceptibility pattern to potassium cyanide when exposed at different times of the day. Polcik et al. [16] later observed variations in efficacy during the day and further proposed that daily changes in the sensitivity towards insecticide treatments are dependent on the properties of the insecticide as well as endogenous rhythmic changes in pest physiology. Subsequently, Halberg et al. [17] suggested that the time of greatest sensitivity is not entirely dependent to a specific moment in the insect’s daily cycle, but also depends on the species, mode of treatment, and/or the insecticide tested. On the basis of these findings, insecticide toxicity measures should therefore be governed by thorough understanding of circadian organization of the detoxification mechanisms.

Circadian rhythms are endogenous, self-sustaining oscillations, which have been described in insects and many other organisms [18–20]. Such rhythms are vital to synchronize crucial life processes with environmental changes, which increase their fitness for survival [21]. To date, circadian rhythms in insecticide susceptibility or chronotoxicity studies have been investigated in insect groups such as mosquitoes [22,23], fruit flies *Drosophila* [24,25], honeybees [26], locusts [27], and cockroaches [28]. Since susceptibility to insecticide can be influenced by the activities of xenobiotic metabolizing (XM) enzymes, these studies also investigated the rhythmic expression of the XM genes and/or their protein products especially Glutathione S-transferase (GST), P450 and esterase. These studies however, are still limited to only a few taxa considering the long history of this discovery, its importance, and the number of pest species around us [29]. Over the years, topical application and residual assays have been the usual parameter of insecticide toxicity evaluation in bed bugs [8,11,30]. Thus far, no information on the chronotoxicity and underlying mechanism of circadian regulation of insecticide susceptibility is available in bed bugs.

Here we investigated the diurnal changes in insecticide sensitivity in *C*. *lectularius* by determining the circadian rhythms in insecticide susceptibility, gene expression of XM genes, and the metabolic enzyme activities. We tested the daily variation of insecticide tolerance in *C*. *lectularius* against two different classes of insecticides, namely pyrethroid which targets the sodium ion channel [31] and neonicotinoid, which acts at nicotinic acetylcholine receptors [32]. Both classes are currently widely used for bed bug control. To understand whether the daily variations in insecticide susceptibility were the physiological consequences of major detoxification enzymes, we investigated the enzymatic activities of P450, Glutathione S-transferase (GST), and esterases, in whole body lysates of *C*. *lectularius*. Subsequently, we selected two genes in CYP family (namely *CYP397A1* and *CYP398A1*), one carboxylesterase (*CE3959*), and one GST (*GSTs1*) to investigate the diurnal rhythmic expressions. These genes have been previously associated with insecticide detoxification metabolism in *C*. *lectularius* [10,33,34].

## Materials and methods

### Bed bugs rearing

An insecticide-susceptible laboratory strain of *C*. *lectularius* (Monheim) was used in this study [35]. Bed bugs were maintained in a climatic chamber (Percival Model CU-41L4, Iowa, USA) under 12-h photoperiod at 28°C, 60–80% relative humidity (RH). The bed bugs were provided blood meals voluntarily by the first author on a weekly basis. Unmated, starved adult male bed bugs, 7 to 10 days after moulting from the last nymphal stage, were used for all experimental assays as unmated adult males have a greater metabolic rate as compared to unmated adult females, and have a stable metabolic rate after feeding between 7 to 10 days [36]. Time of the day reported in this study was set according to Zeitgeber time (ZT), where ZT 0 can be defined as when the lights were on and ZT 12 is when the lights were off. ZT 0 was set at 0700 (UTC+08:00) and ZT 12 was set at 1900 (UTC+08:00). All experiments under the normal 24 h light-dark (LD) cycle were initiated one hour after the lights were switched on (ZT 1). For experiments conducted in 24-hour constant darkness (DD cycle), a Circadian Time (CT) was used to refer to the time unit. All bed bugs were acclimatized in the experiment chamber for two LD cycles prior to the experimental day, to allow the bed bugs to adjust in the new environment [37]. Following acclimatization, The LD experiment was conducted for 24 hours followed by DD experiment for another 24 hours. For each set of experiments (i.e. insecticide assay, biochemical assay, and quantitative real-time PCR), fresh batches of bed bugs were used.

### Insecticide assay

Ten individual adult male *C*. *lectularius* were collected at appropriate ZT and CT time points to demonstrate insecticide susceptibility fluctuation. Deltamethrin, a pyrethroid class insecticide and imidacloprid, a neonicotinoid, were used in this study. The deltamethrin solution was prepared according to the baseline susceptibility data (19.2 mg AI m^-2^) and the imidacloprid solution according to the discriminating rate (192 mg AI m^-2^) suggested in Dang et al. [30]. For deltamethrin assay, 0.5 ml of 0.153 g L^-1^ technical grade deltamethrin (97%, Bayer Environmental Science, Malaysia) solution was decanted on a glass petri dish (70 x 18 mm). Similarly, 0.5 ml of 1.56 g L^-1^ technical grade imidacloprid (95%, Bayer Crop Science, Australia) solution was decanted on a glass petri dish (70 x 18 mm) for imidacloprid assay. The glass petri dishes treated with the insecticides were allowed to dry in a fume hood for an hour prior to insecticide exposure experiment at each time point. Glass petri dish assays were used as suggested by Dang et al. [30]. The cumulative number of insects knocked down was recorded every two minutes for continuous exposure until all the test insects were knocked down. For the control, 10 insects were exposed to the control glass petri dish that was treated with acetone only. An insect was considered to be knocked down if it was unable to move or right itself when gently touched. The knocked down individuals were collected and retained for up to 48 hours in a clean container to observe for mortality. A minimum of three replicates (10 individuals for each replicate) was used for each insecticide in each treatment, and for the controls.

### Biochemical assays

Bed bugs were collected every four hours following the insect circadian cycle at ZT1, ZT5, ZT9, ZT13, ZT17, and ZT21 in the LD cycle and at CT1, CT5, CT9, CT13, CT17, and CT21 of the DD cycle. They were homogenized with a micropestle in 600 μl of ice-cold 0.1M phosphate buffer (pH 7.0) for protein extraction. The homogenate was then centrifuged at 10,000g for 5 min and the supernatant collected. Total protein concentration was determined using the Bradford protein concentration assay [38], and bovine serum albumin (BSA) was used as a standard protein. The absorbance was read at 570 nm after 5 min incubation at 22°C. Five biological replicates (one individual per replicate) were conducted with five technical replicates for every assay tested. All assays were measured using the microplate reader SYNERGY MIX (Bio-Tek Instruments, USA).

#### Glutathione S-transferase (GST) assay

The assay activity was determined using the substrate 1-chloro-2,4-dinitrobenzene (CDNB), following the method described in Karunaratne et al. [39]. 10 μl of homogenate was mixed with 200 μl of substrate solution (190 μl of 10.5Mm reduced glutathione in 100mM phosphate buffer and 10 μl of 63mM of CDNB in methanol). The plate was immediately read using a microplate reader at 340 nm, with measurements taken every minute for five minutes. Blank samples without the homogenate were also included in every run. An extinction coefficient of 5.76 mM^-1^ with corrected path length of 0.6 cm was used. The increasing rate of absorbance of GS-DNB conjugate was calculated as GSTs activity, and the unit was reported as nmol/min/mg.

#### Esterase assay

General esterase activity was adapted from Yoon et al. [40] with a slight modification; alpha-naphtyl acetate (α-NA) and beta-naphtyl acetate (β-NA) were used as the substrates. 50 μl of α-NA (0.5 M final concentration) or β-NA (0.5 M final concentration) were mixed with 10 μl homogenate and 40 μl of 0.1 M phosphate buffer (pH7.0) in a microplate. The microplate was incubated for 30 min at 30°C in the microplate reader. 50 μl of freshly prepared stop solution (22.5 mg Fast blue B in 2.25 ml of distilled water and 5.25 ml of 5% sodium lauryl sulfate diluted in 0.1 M phosphate buffer, pH 7.0) was added and the mixtures were incubated for two minutes and read at 600 nm and 560 nm for α-NA and β-NA respectively. The absorbance values were determined based on the production of α- and β- naphthol.

#### P450 assay

The P450 assay was carried out according to methods described in Yoon et al. [40] by using 7-ethoxycoumarin (7-EC) as a substrate to determine the cytochrome P450-dependent O-deethylation activity (ECOD). 10 μl of homogenate was mixed with 2 μl of 20 mM 7-EC and 80 μl 0.1 M phosphate buffer (pH 7.0). Then, 10 μl of 10 mM NADH solution in 0.1 M phosphate buffer (pH 7.0) was added to initiate the reaction. The mixtures were incubated at 30°C for 20 min and agitated every 5 min during the incubation. The remaining NADH was removed by adding 10 μl oxidized glutathione (10 mM, final concentration) and 1 unit of glutathione reductase (Sigma Aldrich). After 10 min incubation at room temperature, 125 μl of a solution containing 50% acetonitrile in Trizma-base buffer (0.05M, pH 10) was added to stop the reaction. The fluorescence was measured after 5 min using a microplate fluorescent reader at the emission wavelength of 465 nm and the excitation wavelength of 390 nm.

### Quantitative real-time PCR (qRT-PCR)

Adult male bed bugs were collected at each time points, as mentioned previously. Total RNA was isolated from the adult bed bug by using RNeasy Mini Kit (Qiagen, Germany) and treated with DNase I (Qiagen, Germany). Subsequently, cDNA was synthesized using QuantiNova Reverse Transcription Kit (Qiagen, Germany). Daily fluctuation expressions of the XM genes as inferred from Zhu et al. [10] were measured by RT-PCR with gene specific primers (Table 1). For genes coding for carboxylesterases, we initially chose *CE3959* and *CE21331* which are significantly upregulated in resistant bed bug strains [10,33]. Nonetheless, *CE21331* fails to produce reliable PCR amplifications across the samples tested, therefore only *CE3959* was used in the current study. Normalization of genes was calculated relative to ribosomal protein L18 (*rpl* 18), which is the most stable housekeeping gene for *C*. *lectularius* across different developmental stages and tissue samples as described in Mamidala et al. [41]. All primers tested in this study had a PCR efficiency of >90%. PCR efficiency is particularly important when reporting mRNA concentrations for target genes relative to those of reference genes. The genes must be amplified with comparable efficiencies for this comparison to be accurate. The slope of the log-linear portion of the calibration curve is used to calculate the PCR efficiency of the assay [42]. Each qRT-PCR reaction (10μl final volumes) contained 5μl QuantiNova SYBR Green PCR Master Mix (Qiagen, Germany), 0.25 μl cDNA (10 ng), 3.75 μl ddH_2_O, and 0.5 μl each forward and reverse gene specific primers. The PCR conditions were set as follows: 95°C for 5 min followed by 40 cycles at 95°C for 10 s, 60°C for 30 s, 72°C for 30 s, and finally 72°C for 10 min. The reaction was concluded with a melt curve analysis beginning from 60°C to 95°C with 0.1°C increment every 5s per step. All qRT-PCR were performed in StepOnePlus Real-Time PCR System (Applied Biosystems, USA) and non-template control reaction was run in each assay to avoid nucleic acids contamination. Real-time quantitative data were analysed by using the standard 2^-ΔΔC^_T_ method [43]. Value of 2^-ΔΔC^_T_ was derived from calculation ΔΔC_T_ = (CT, Target−C_T, *rpl18*_) _Time_x–(C_T,Target_−C_T,*rpl18*_)_Time ZT1_. All calculations for relative gene expressions (fold-change) were determined relative to ZT1 time point. Three biological replicates, each with four technical replicates were used for each gene expression assay at each time point.

**Table 1:** Primers used in qRT-PCR.

Gene	Forward primer (5’– 3’)	Reverse primer (5’– 3’)	PCR efficiency (%)	Amplicon size (bp)
Housekeeping gene				
*rpl18*	AAAGGCACGGTTACATCAAAGGTG	TAGTCTTGAACCTATAGGGGTCCC	96.88	150
Target gene				
Glutathione S- tranferase				
*GSTs1*	AGGAGAGCCAGTTAGATTTATGTT	AAGCGATTCCCACCGATTTT	97.28	250
Cytochrome P450				
*CYP397A1*	TATTGGAGTCGACAGGGCGTGAAA	TGACATCGCCCAATTGCTTGTAGC	95.32	150
*CYP398A1*	TGTCGACCCAATGATGGCTCTGAA	GAAATTGGAGGCCGATTTGGCGAT	92.43	190
Carboxylesterase				
*CE3959*	ACGTCTGGAGAAGGGCAACTGAAA	GACGGCCGGGTAGATGAAAACAAC	91.86	250

### Statistical analysis

For insecticide assays, the knockdown data were pooled and subjected to probit analysis [44], after correcting control knockdown using Abbott’s formula [45]. The goodness-of-fit test χ² test was used to affirm that the dataset met the assumptions of the probit model. Time taken to achieve knockdown of 50% of test insects exposed to a fixed dose of insecticides, KT_50_ values, were determined from the probit analysis by using SPSS v20 (IBM SPSS Inc., 2012). The KT_50_ values were considered significantly different (*P*<0.05) when their 95% confidence intervals (CIs) did not overlap [46,47]. The survival time data at different ZT and CT periods were analysed using survival analysis (log-rank test) in SPSS v20. The distribution of survival time was described using the survivorship function S(t). Kaplan-Meier survival curves were generated. Significance tests for time-of-day differences in gene expression data (ΔC_T_ values), and enzyme metabolic assays were determined based on analysis of variance (one-way ANOVA) and Tukey’s pairwise comparison (*P*< 0.05) generated from SPSS v20. CircWave v1.4 software (www.huttlab.nl, an extension of cosinor analysis, courtesy of Dr. Roelof Hut, www.euclock.org) was used to analyse the rhythmicity of gene expression, total enzyme activity, and insecticide bioassays fitting a Fourier-curve (one sine wave plus up to one additional harmonic) to the data [48]. Significance was set at alpha = 0.05 and Bonferroni adjustment was made to account for Type I error for multiple comparisons [49].

## Results

### Circadian rhythms in *C*. *lectularius* susceptibility to insecticides

**Fig 1:**
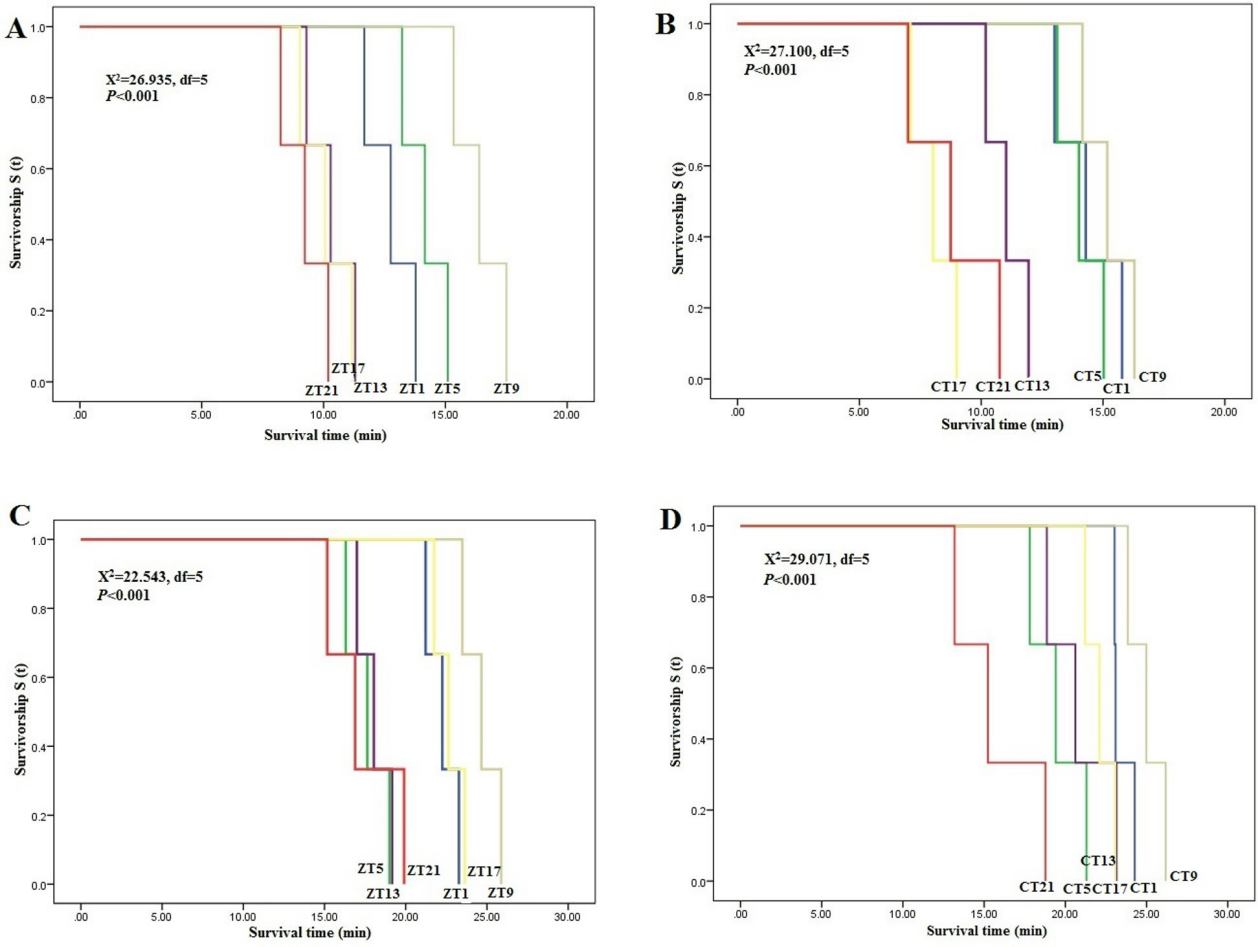
Kaplan-Meier survival analyses for the susceptible Monheim strain of *C*. *lectularius* when exposed to deltamethrin and imidacloprid. (A) Survival time in LD condition when exposed to deltamethrin. (B) Survival time in DD condition when exposed to deltamethrin. (C) Survival time in LD condition when exposed to imidacloprid. (D) Survival time in DD condition when exposed to imidacloprid. Survivorship function S (t) was defined as the probability that an individual survives longer than time “t”.

All of the knocked down individuals did not regain consciousness after 48 hours of observation indicating the knocked down individuals resulted in eventual mortality. Susceptibility to insecticides was assessed based on the KT_50_ value. The results showed that there was a significant difference in the knockdown time between the observed test points in both LD and DD conditions for both deltamethrin and imidacloprid bioassays (Table 2). In deltamethrin bioassay, the *C*. *lectularius* displayed longest KT_50_ value during the day in LD conditions at ZT9 (Table 2). A similar pattern was observed in the DD condition with the longest KT_50_ values were observed in the subjective day of the DD cycle (Table 2) at CT9. Cosinor analysis revealed that daily fluctuation to deltamethrin susceptibility were rhythmic (*P*< 0.0083) in both the LD (*R*^2^ = 0.783, *P*< 0.0083) (S1A Fig) and DD (*R*^2^ = 0.8413, *P*< 0.0083) (S1B Fig) conditions. Similarly, longest KT_50_ values were observed at ZT9 and CT9 of the LD and DD cycles of imidacloprid bioassay, respectively (Table 2). Nonetheless, cosinor analysis revealed no diel rhythms in the daily fluctuation to imidacloprid susceptibility in both LD and DD conditions (S1C and S1D Fig). The Kaplan-Meier survival curve analysis revealed that the bed bugs have a consistent longer survival time in the photophase than the scotophase of the LD condition (Fig 1A) and similarly throughout the subjective day of the DD condition (Fig 1B) when challenged with deltamethrin. Under both conditions, survival increases towards the end of photophase at ZT9 in LD and at CT9 of DD conditions. For imidacloprid, survival times were found alternating at each ZT/CT intervals in LD (Fig 1C) and DD (Fig 1D) conditions, respectively.

**Table 2:** KT_50_ of bed bugs for the susceptible Monheim strain of *C*. *lectularius* exposed to deltamethrin and imidacloprid at different conditions and time points.

Insecticides	Condition	Time point	N	KT50 (95% CI)(min)^*^
Deltamethrin	LD (ZT)	1	30	12.761 (11.665–13.788)^a^
		5	30	14.156 (13.218–15.109)^a^
		9	30	16.392 (15.342–17.515)^b^
		13	30	10.293 (9.296–11.302)^c^
		17	30	10.067 (9.027–11.173)^c^
		21	30	9.230 (8.239–10.191)^c^
	DD (CT)	1	30	14.289 (13.014–15.782)^a^
		5	30	14.011 (13.017–15.027)^a^
		9	30	15.176 (14.156–16.289)^a^
		13	30	11.024 (10.184–11.945)^b^
		17	30	8.024 (7.095–8.993)^c^
		21	30	8.746 (6.993–10.757)^bc^
Imidacloprid	LD (ZT)	1	30	22.251 (21.224–23.277)^a^
		5	30	17.633 (16.305–19.001)^b^
		9	30	24.656 (23.482–25.875)^c^
		13	30	18.039 (16.991–19.161)^b^
		17	30	22.632 (21.743–23.633)^ac^
		21	30	16.884 (15.166–19.899)^b^
	DD (CT)	1	30	23.089 (23.035–24.273)^a^
		5	30	19.406 (17.812–21.305)^b^
		9	30	24.982 (23.846–26.178)^a^
		13	30	20.619 (18.858–23.153)^ab^
		17	30	22.101 (21.218–23.069)^ab^
		21	30	15.233 (13.181–18.775)^b^

^*^Different letters in the table indicate significant differences based on the 95% confidence interval.

### Circadian rhythms in enzymatic activity of whole *C*. *lectularius* lysates

**Fig 2:**
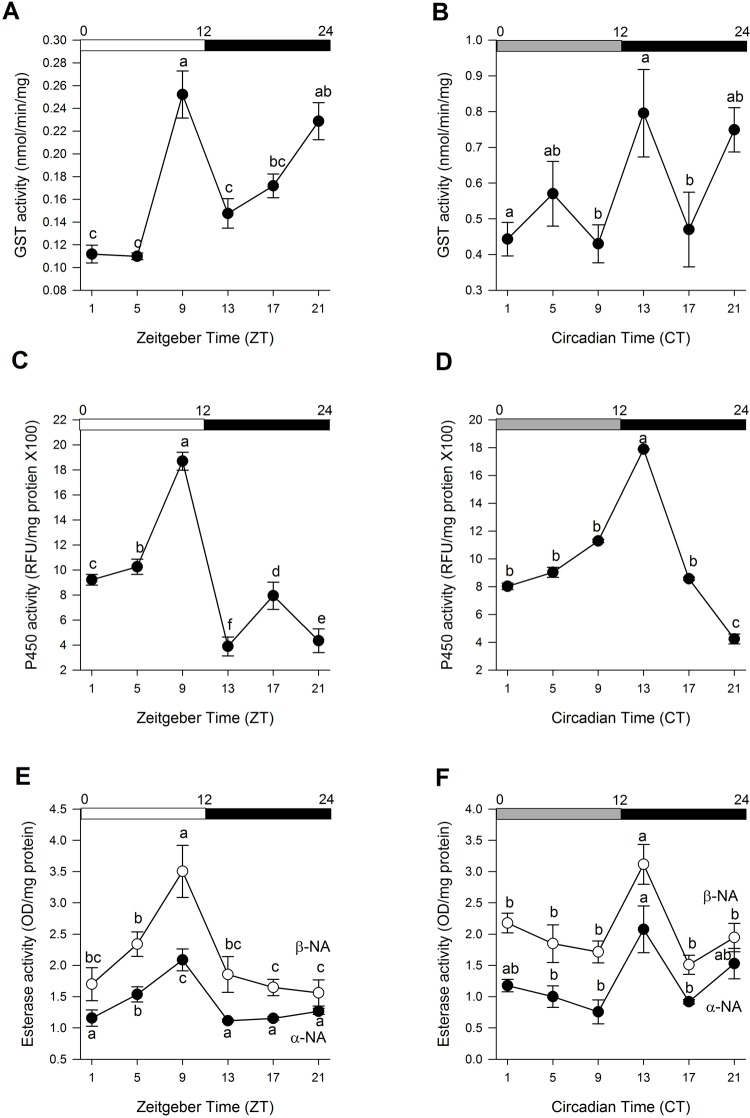
Daily fluctuation of enzyme activities in *C*. *lectularius* throughout the day at LD condition and DD condition. (A–B) Glutathione S-transferase enzyme activity. (C–D) P450 enzyme activity. (E–F) General esterase enzyme activities. Data represent mean +/- SEM (n = 5 for five replicates). Different letters indicates a significant difference (One-way ANOVA and Tukey’s analysis, *P*<0.05). White and dark bars above denote day and night time respectively, while grey bay indicate subjective day in DD condition.

Biochemical assays were performed on whole *C*. *lectularius* lysates collected every 4 hours under LD and DD conditions to determine the total GST, esterase, and CYP450 activity. The enzymatic assay for each GST, esterase, and P450, revealed significant time-of-day specific changes (_ANOVA,_ *P*< 0.05; Fig 2). All three enzymes tested have similar peak phases with the highest activity consistently recorded at ZT9 in LD conditions. Furthermore, these enzyme activity patterns persisted under constant dark conditions, suggesting that they are potentially driven by an endogenous circadian clock. Enzyme activity progressively increased in the photophase towards mid-day before subsiding in the scotophase. Esterase activities using two different substrates α-NA and ß-NA (Fig 2E and 2F) resulted in similar pattern indicating consistency and efficiency of the enzyme assays. In addition, all the enzymes displayed the same peak activity during constant darkness at CT13. Cosinor analysis revealed that the enzymatic activity of P450 was rhythmic in both LD (*R*^2^ = 0.6523, *P*< 0.0083) and DD (*R*^2^ = 0.983, *P*< 0.0083) conditions (S2C and S2D Fig). On the other hand, GST (*R*^2^ = 0.8816, *P*< 0.0083) (S2A Fig) and esterase (α-NA: *R*^2^ = 0.9789, *P*< 0.0083; ß-NA: *R*^2^ = 0.8404, *P*< 0.0083) (S2E Fig) activities were found rhythmic in only LD conditions.

### Circadian rhythms in metabolic gene expression

**Fig 3:**
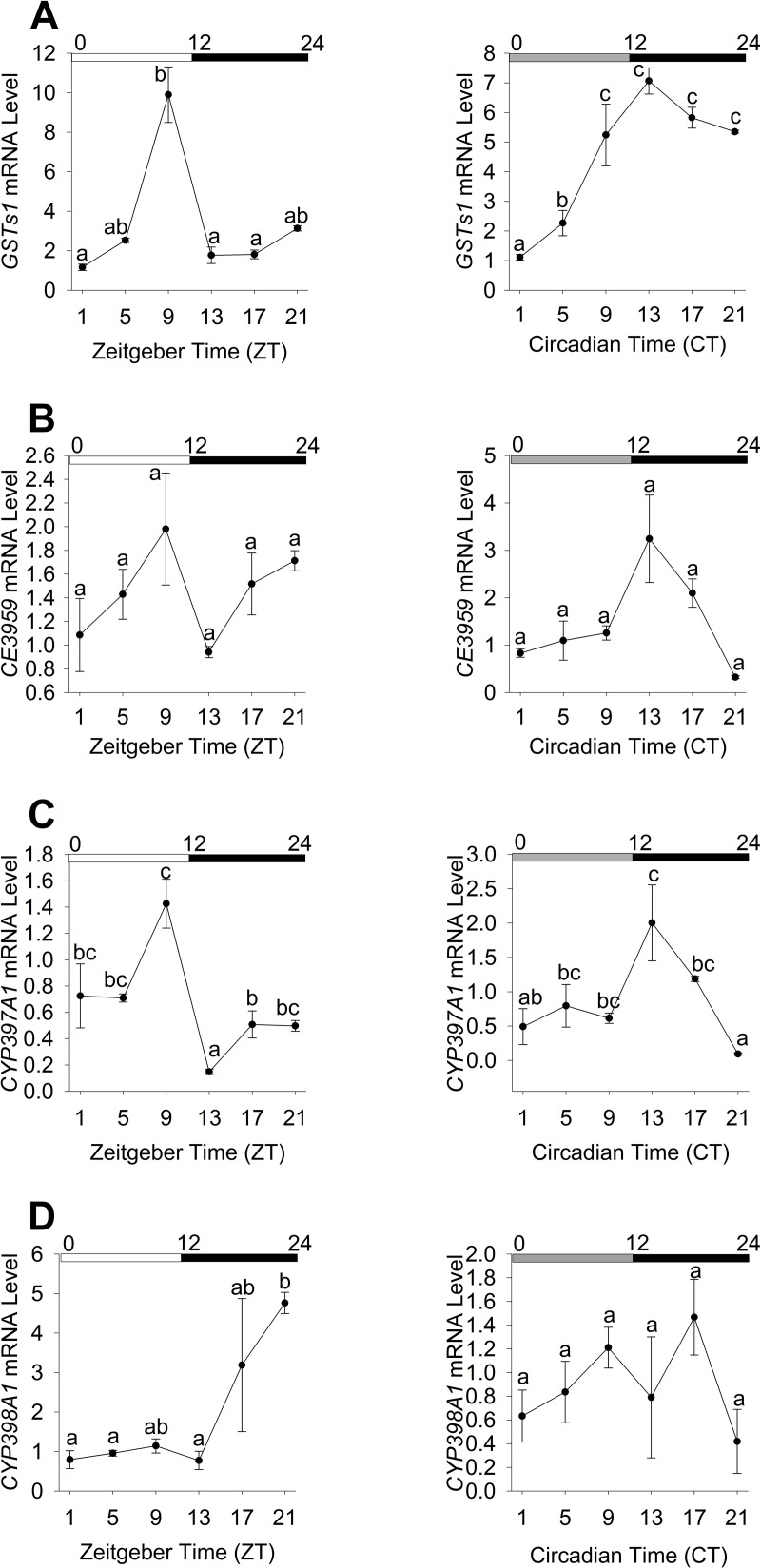
Daily mRNA expression of detoxification genes in *C*. *lectularius* under LD and DD conditions. (A) *GSTs1* mRNA expression level. (B) *CE3959* mRNA expression level. (C) *CYP397A1* mRNA expression level. (D) *CYP398A1* mRNA expression level All data were normalized using mRNA levels of *rpl18* and relative to ZT 1. Data represent mean +/- SEM of three replicates. Different letters on the graph indicate significant differences (One-way ANOVA and Tukey’s test, *P*<0.05). White and dark bars above denote day and night time respectively, while grey bar indicates subjective day in DD condition. ZT and CT are abbreviations for time in light dark cycle and constant darkness respectively.

The mRNA expression levels of four selected XM genes in *C*. *lectularius* were determined based on real-time PCR under LD and DD conditions. One-way ANOVAs revealed significant time-of-day differences in mRNA expression patterns of *GSTs1*, *CYP397A1*, and in both LD and DD conditions (Fig 3). However, *CYP398A1* was only significant in LD condition (Fig 3D) whereas there were no significant differences observed within the LD and DD phases of *CE3959* (Fig 3B). A circadian expression pattern was found for *GSTs1* (Fig 3A) and *CYP397A1* (Fig 3C). Both these genes showed a similar expression pattern, with increasing mRNA expressions during the photophase in LD conditions with peak expression in late photophase at ZT9 before the mRNA expressions decreased in the scotophase. This pattern was similar in the constant dark (DD condition) except a shift in the peak expression at CT13 instead of CT9 as observed in the LD condition. *CYP398A1* expression patterns were inconsistent with low level of expression in the presence of light and progressively elevated throughout the dark phase. Cosinor analyses revealed that expression patterns were rhythmic for *GSTs1* (LD: *R*^2^ = 0.8353, *P*< 0.0083; DD: *R*^2^ = 0.8553, *P*< 0.0083) (S3A and S3B Fig) in LD and DD conditions. On the other hand, expression patterns of *CE3959* (*R*^2^ = 0.7207, *P*< 0.0083) (S3D Fig) and *CYP397A1* (*R*^2^ = 0.6786, *P*< 0.0083) (S4B Fig) were rhythmic only in DD, whereas *CYP398A1* was only rhythmic in LD (*R*^2^ = 0.6765, *P*< 0.0083) (S4C Fig).

## Discussion

Biological rhythms are manifested in various life processes such as locomotor activity [37], feeding behaviour [26], sleep patterns [50], and various other physiological and metabolic pathways [51]. In this study, we present analyses of circadian rhythms in insecticide susceptibility of *C*. *lectularius* against deltamethrin and imidacloprid that belongs to two different classes of insecticides. We also investigated the temporal expression of genes coding insecticide metabolizing enzymes, and the activity of these enzymes in the common bed bug, *C*. *lectularius*. Our investigation showed that the *C*. *lectularius* displayed circadian rhythm in insecticide susceptibility to deltamethrin, but this condition was not observed in the imidacloprid bioassay (Fig 1). Cosinor analysis in the present study determines the rhythmicity at 24 hours wave length. A cycle with shorter period is possible for imidacloprid; however, the identification of such rhythm is limited under current analysis. The difference in mode of action between the two classes of insecticides that act at different target sites on the insect neuron may have contributed to the difference in observations. Pyrethroids act by interfering with sodium ion channels along the nerve axon [31], whereas neonicotinoids bind to nicotinic acetyl choline receptors on the postsynaptic membrane [32]. Similar findings have been observed in other insects involving exposure to various classes of insecticides such as in *Drosophila suzukii* Matsumura whereby significant daily undulation of insecticide susceptibility was observed against the organophosphate (malathion) but absent against pyrethroids (fenpropathrin) [25]. Hooven et al. [24] also reported significant time-of-day specific changes in insecticide susceptibility of *Drosophila melanogaster* Meigen against an organophosphate (malathion), a carbamate (propoxur), and a phenylpyrazole (fipronil), but no rhythmic pattern of daily susceptibility was found against a pyrethroid (permethrin). These findings therefore suggest species specific differences in daily rhythms of insecticide susceptibility to different classes of insecticides. In the present study, we found that *C*. *lectularius* is most tolerant of deltamethrin during the middle of the day, approximately nine hours (4pm) after light is present (Table 2). Further investigation of the chronotoxicity in the constant darkness resulted in similar peak phases, which suggest an endogenous circadian clock underlying this rhythmic susceptibility to deltamethrin. This result is consistent with previous published works that associated circadian control of pyrethroid susceptibility in *B*. *germanica* [28] and *Aedes aegypti* L. [22] although peak activity to insecticide exposure varies from one insect to another. *Cimex lectularius* displayed peak survival during the photophase of the LD condition, which is similar to that found in *Ae*. *aegypti* (ZT9 for permethrin) [22], in *Anopheles gambiae* Giles (ZT10.6 for DDT, and ZT0.6 and ZT 10.7 for deltamethrin) [23] and in the silkworm, *Bombyx mori* L. (ZT9 for permethrin) [52]. Whereas the opposite (i.e. peak survival during the scotophase) was observed in *B*. *germanica* (ZT21 for permethrin) [28] and in *D*. *suzukii* (ZT 20 for malathion) [25].

In most biological systems, detoxification pathways have evolved to aid the metabolism of toxic chemicals that an organism may contact from the environment through expression of the enzymes involved in their metabolism. To determine whether circadian rhythms in insecticide susceptibility were the physiological consequences of major detoxification enzymes, we investigated the enzymatic activities of P450, GST, and esterase, known to be involved in metabolic resistance in bed bugs. The expression patterns of all three enzymes tested were consistent with the circadian rhythm observed in deltamethrin assay with highest activity recorded at ZT9 in LD condition, suggesting the potential role of these enzymes in mediating daily variations to deltamethrin susceptibility in *C*. *lectularius*. Based on these findings, we subsequently investigated the gene expression patterns of candidate XM genes from each enzyme families. The XM genes such as CYP, GST, esterase, and UDP-glucuronosyl-transferase (UGT), are highly conserved in insects [53]. The circadian oscillation of XM genes that has been documented to influence the susceptibility of insects against insecticides, has been observed in several insects including *Ae*. *aegypti*, *An*. *gambiae*, and *D*. *melanogaster* [54–56]. The *C*. *lectularius* genome contains 58 genes and one pseudogene coding for P450 enzymes, 30 carboxylesterase genes and 12 GST genes [57]. Among them, several candidate XM genes have been associated with insecticide resistance based on transcriptomic evidence, gene expression and/or RNA interference. Adelman et al. [33] reported that there is a link between insecticide resistance and constitutive over expression of P450 genes, namely *CYP397A1*, *CYP6DM2*, and *CYP400A1*, in *C*. *lectularius*. In addition, four P450 genes, *CYP9* [58], *CYP397A1V2*, *CYP6A2*, and *CYP6A13* [34], were linked to *C*. *lectularius* resistance to pyrethroids. Subsequently, Zhu et al. [10] confirmed the involvement of four P450 genes; *CYP397A1*, *CYP398A1*, *CYP6DN1*, and *CYP4CM1*, to be involved in *C*. *lectularius* resistance to pyrethroids, based on the dsRNA-mediated interference (RNAi) approach. Adelman et al. [33] also reported that two carboxylesterase encoding genes *CE3959* and *CE21331* to be significantly upregulated in the pyrethroid resistant *C*. *lectularius* Richmond strain. Zhu et al. [10] also found that the gene *CE21331* was associated with *C*. *lectularius* pyrethroid resistance, due to over expression in resistant strains with maximum up-regulation of >50-fold among the field populations tested. Similarly, Adelman et al. [33] identified one candidate GST gene *GSTs1* to be putatively involved in mediating resistance to pyrethroids in *C*. *lectularius* based on significant up-regulation in the pyrethroid resistant Richmond strain. Based on these reports, we selected candidate XM genes from each gene families following their significant high expression levels in the resistant strains and/or functional validation through RNA interference that suggest their potential involvement in the circadian variation of susceptibility to insecticides in bed bugs.

The data reported in our study suggest a potential link between the circadian rhythms in the expression of genes coding the metabolic enzymes and insecticide susceptibility against deltamethrin. The mRNA expression patterns of *GSTs1* and *CYP397A1* were similar to the susceptibility pattern observed in deltamethrin toxicity assay with the highest peak expression reported at ZT9 in LD condition. However, only *GSTs1* was rhythmic in both LD and DD conditions and therefore likely to be under circadian regulation, which is congruent with the observations from deltamethrin assay. An association between rhythmic expression of genes coding metabolic enzymes and rhythmicity towards insecticide susceptibility has also been reported in few other studies. In mosquitoes for instance, Yang et al. [22] reported a positive link between *CYP9M9* and permethrin susceptibility in *Ae*. *aegypti* whereas Balmert et al. [23] reported a correlation between *CYP6M2*, *CYP6P3*, and *CYP6Z1* with permethrin susceptibility in *An*. *gambiae*. Similarly, such correlations were also observed in other insects such as *D*. *suzukii* (*GSTD2*, *GSTD7*, *a-est7*, *CYP6g1*, *CYP6a2* with organophosphates) [25] and *B*. *germanica* (*GSTD1* with pyrethroids) [28].

Rhythmic mRNA expression levels are likely to correspond to rhythmic gene protein abundance. In our study, we found that the gene expression patterns of *GSTs1* and *CYP397A1* corresponded similarly with the daily fluctuations in total enzyme activities of GST and P450, respectively. The enzyme activities and gene expressions consistently displayed the highest peak activity at ZT9 in LD condition. The rhythm persists in the absence of external cue (Zeitgeber) in DD condition. Nonetheless, the absence of any environmental cues often results in a free-running period that does not correspond perfectly to the period of the environmental rhythm. In the present study, we observed a consistent phase delay in peak expressions of *GSTs1* and *CYP397A1* and the respective enzyme activities in DD condition (free-running period) whereby peak activity was observed at CT13. Persistent of this rhythm and occurrence of phase shift in the free-running period provided evidence for the existence of circadian mechanism underlying such activity. Nonetheless, further experiments targeting the disruption of clock genes and finding their association with the genes studied here is necessary to provide empirical evidence of circadian control of endogenous genes in mediating circadian rhythms in insecticide susceptibility in bed bugs.

The gene expression of *CE3959* however did not correspond to the rhythmic expression of total esterase activity. In the present study, the broad enzymatic assays were not specific to enzymes implicated in the breakdown of the insecticides. Instead, the assays detect total oxidase, GST and esterase activities from a broad range of enzymes from the respective enzyme group. As seen in Fig 3C and 3D, rhythmic peaks in specific CYPs occurred at different phases in the LD and DD conditions. Thus, it is not surprising that the *CE3959* expression does not correspond to total esterase activity; suggesting that other candidate carboxylesterase genes could be related to the observed total esterase activity [57].

Since bed bugs are a nocturnal insect, increased deltamethrin susceptibility and reduced metabolic detoxification activities are expected to coincide with the inactive phase of daily activity as animals tended to have higher possibilities of encountering xenobiotic stress during their active phase such as feeding and mate seeking. Such positive association has been observed in other insect groups such as in the nocturnal pine weevils *Hylobius abietis* L. [29], in the diurnal *Ae*. *aegypti* [22], and in *Musca domestica* L. that shows positive association between an increase in food consumption activity to an increase in insecticide tolerance [59]. Romero et al. [37] reported that *C*. *lectularius* displayed highest levels of locomotor activity during dark hours across all mobile stages which reflected the nocturnal nature of the species. Our findings showed that *C*. *lectularius* consistently displayed increased metabolic detoxification activity and tolerance to insecticide towards late photophase. This finding suggests that the bed bugs would upregulate XM genes in late photophase in preparation or anticipation of later feeding in the early scotophase. Xenobiotic metabolic detoxification processes in bed bugs may also involve different enzyme candidates with different active phases, such as ABC-transporters, *Abc8* and *Abc9* previously associated with insecticide resistance in bed bugs [10], which are yet to be explored.

Overall, our results have demonstrated the consequence of biological timing to the regulation of genes involved in metabolic detoxification and sensitivity to insecticidal challenge. Although the present study suggests that *C*. *lectularius* is most vulnerable to deltamethrin at dawn and dark hours where bed bugs are most active and therefore have higher chances in contacting the insecticide, the differences in KT_50_ values between the time points are, small. Such small differences (yet significant) may be caused by the insecticide susceptible Monheim strain used in the study. Similar observation was also seen in another study using the insecticide susceptible Mali strain of *An*. *gambiae* [23] whereby the KT_50_ values ranged within 30 mins and 15 mins across the 24 hours observations when tested against DDT and deltamethrin, respectively. A comparative study against field collected and insecticide resistant strains of bed bugs may give a better overview of the extent of daily susceptibility pattern observed in this study. Nonetheless, the high level and multiple modes of resistance mechanisms to insecticide in the field collected strains would make performing physiologically relevant time-of-day specific assays challenging, especially in determining an appropriate discriminating dose of the insecticide to achieve mortality within the 24 hour time frame [10]. To this extent, this study has provided a fundamental basis for the chronotoxicity research in bed bugs and has provided preliminary evidence on molecular mechanisms underlying the diurnal variation of insecticide susceptibility in bed bugs which was previously unknown. Knowing time-of-day susceptibility to insecticide can be useful to enhance success of insecticide based treatment regimens. In the case of bed bugs, the pyrethroids and neonicotinoids both provide poor residual control against resistant strains [5,60,61]. Topical application is more efficacious, and perhaps application timing could result in better control with resistant strains. The application timing also varies from insects to insects and between different classes of insecticides. Our findings also suggest that the standard testing of insecticides and reporting of efficacy data should take into considerations of the biological timing as this will allow reliable estimation of insecticide efficacy and reproducibility across other studies.

## Supporting information

S1 Fig Cosinor analyses for the susceptible Monheim strain of *C*. *lectularius* when exposed to deltamethrin and imidacloprid at LD and DD conditions. (TIFF)

S2 Fig Cosinor analyses for enzyme activities of *C*. *lectularius* at LD and DD conditions. (TIFF)

S3 Fig Cosinor analyses for daily expression of detoxification gene expression (*GSTs1* and *CE3959*) in *C*. *lectularius* at LD and DD conditions. (TIFF)

S4 Fig Cosinor analyses for daily expression of detoxification gene expression (*CYP397A1* and *CYP398A1*) in *C*. *lectularius* at LD and DD conditions. (TIFF)
